# Innocent parties or devious drug users: the views of primary healthcare practitioners with respect to those who misuse prescription drugs

**DOI:** 10.1186/1477-7517-7-21

**Published:** 2010-09-26

**Authors:** Rachael Butler, Janie Sheridan

**Affiliations:** 1 School of Pharmacy, University of Auckland, Private Bag 92019, Auckland, New Zealand

## Abstract

**Background:**

Many health professionals engage in providing health services for drug users; however, there is evidence of stigmatisation by some health professionals. Prescription drug misusers as a specific group, may also be subject to such judgment. This study aimed to understand issues for primary care health practitioners in relation to prescription drug misuse (PDM), by exploring the attitudes and experiences of healthcare professionals with respect to PDM.

**Methods:**

Tape-recorded interviews were conducted with a purposive sample of general practitioners (17), community pharmacists (16) and 'key experts' (18) in New Zealand. Interviews were transcribed verbatim and a thematic analysis undertaken. Participants were offered vouchers to the value of NZ$30 for their participation.

**Results:**

A major theme that was identified was that of two different types of patients involved in PDM, as described by participants - the 'abuser' and the 'overuser'. The 'abuser' was believed to acquire prescription medicines through deception for their own use or for selling on to the illicit market, to use the drugs recreationally, for a 'high' or to stave off withdrawal from illicit drugs. 'Overusers' were characterised as having become 'addicted' through inadvertent overuse and over prescribing, and were generally viewed more sympathetically by practitioners. It also emerged that practitioners' attitudes may have impacted on whether any harm reduction interventions might be offered. Furthermore, whilst practitioners might be more willing to offer help to the 'over-user', it seemed that there is a lack of appropriate services for this group, who may also lack a peer support network.

**Conclusions:**

A binary view of PDM may not be helpful in understanding the issues surrounding PDM, nor in providing appropriate interventions. There is a need for further exploration of 'over users’ whose needs may not be being met by mainstream drug services, and issues of stigma in relation to ‘abusers’.

## Background

The use of drugs within society is an emotive issue and continues to garner much attention, politically, socially and within the media. Different drugs, however, are likely to evoke distinct responses depending on their legal status, the perceived level of harm, and - ultimately - how acceptable they are considered within mainstream society. As Room notes in his discussion on stigma [1], social inequality and alcohol and drug use, "psychoactive substance use occurs in a highly charged field of moral forces" (p.152). He claims that at least one aspect of their use usually attracts marginalisation and stigma for the consumer involved. This may be to do with moral judgments regarding intoxication, or due to state sanctions of drug-using members of society. However, substance use can, in some cases, be viewed in a more accepting and indeed aspirational fashion - and Room cites examples such as complementary drinks in prestigious settings, or ecstasy use in some youth subcultures [1].

Prescription drugs (or pharmaceuticals) - and their misuse - are an interesting case in point. These are legally available substances distributed by healthcare practitioners in the treatment of medical conditions and are seen to be a legitimate form of substance use, given their regulations and controls. It is widely recognised, however, that prescription medicines are liable to abuse/misuse and the issue has received increasing attention from governments and policy makers in recent years [2-4]. An increase in illicit use of these substances has been attributed to their perceived 'safe' image (particularly compared with illegal street drugs) and their increasing availability [5]. Moreover, their 'reliability' compared to illicit street drugs, where the quality and dose of the drug may not be known, has been highlighted as an attractive feature to drug users [6]. In New Zealand (NZ), drugs such as cocaine and heroin are expensive and not widely available [4], and it is hypothesised that pharmaceuticals thus feature highly within New Zealand's illicit drug markets [4]. Data collected from frequent drug users on an annual basis via the NZ Illicit Drug Monitoring System (IDMS) gives us some insight into trends with regard to these substances. Recent results, for example, illustrate some key differences between the availability of 'street' or illicit morphine versus heroin. Of note, over two thirds of 'frequent drug users' claimed that they would be able to purchase supplies of street/illicit morphine in an hour or less, whilst less than half said they would be able to source heroin in the same time frame [7]. To date, little research exists on the views of healthcare practitioners towards those who misuse prescription medicines.

In New Zealand, patients pay a fee to see their general practitioners (GPs), as well as paying a fixed price for their medicines when these are dispensed by community pharmacists (CP) (if these are subsidised by the government). At the time of the study a prescription charge could be between $3 and $15 per item, and a visit to a GP could have costs of up to $80 per visit, although normally this would be considerably less. This paper will explore how GPs and community pharmacists CPs in New Zealand, when being interviewed about prescription drug misuse and its impact on primary care practice, 'classified' prescription drug misusers, and how this influenced their response to such patients, including whether or not any kind of harm reduction intervention was offered. As a part of this, we explore the cultural meanings surrounding prescription drug misuse, and the different notions of 'good' and 'bad' qualities ascribed to patients involved in this behaviour by their primary healthcare practitioners. This paper forms part of a larger study. A copy of the report may be seen at: http://www.ndp.govt.nz/moh.nsf/pagescm/7540/$File/prescription-drug-misuse-primary-care-2008v2.pdf

## Methods

This study involved qualitative, semi-structured interviews with primary healthcare practitioners and other 'key experts'. Sampling for both groups was purposive. This approach seeks to select individuals based on their knowledge, experience or specific characteristics [8]. In the context of this study, GP and CP interviewees were selected in consideration of their gender, length of time practising, the location of their practice or pharmacy (i.e. rural vs. urban locale) and whether or not they dispensed or prescribed methadone. All these factors were considered potential influences on their views and experiences as a primary healthcare practitioner (PHCP) with regard to prescription drug misuse. 'Key experts' (KEs) were selected for their specialist knowledge in areas relevant to the research including drug treatment, and law enforcement.

A mix of telephone and face-to-face interviews were conducted between June 2007 and January 2008. Participants were provided with a NZ$30 voucher in recognition of their time. With the permission of the research participants, interviews were recorded on a digital device and later transcribed verbatim. A thematic analysis of the data, employing a general inductive approach [9], was carried out. The NVIVO software package was utilised during the analysis process to assist with the coding and management of the data.

For the purpose of this study the following definition of prescription drug misuse was utilised in the Participant Information Sheet: "*You are invited to take part in a study which is exploring the diversion and misuse or abuse of prescription drugs by patients/clients. A definition of this type of drug misuse/abuse is the misuse or illicit acquisition or diversion of prescription drugs for their psychoactive effects. Although not all prescription drugs obtained for this purpose are sourced through GPs or dispensing pharmacists, accessing them via primary care is thought to be a significant source. This is, therefore, the focus of this piece of research"*. This definition was developed by the project advisory group and is in line with that used by Weekes et al [10]. A verbal explanation of this was given by the researcher at the beginning of each interview, and any misunderstandings clarified. The rationale was to include only psychoactive medicines with abuse/addiction potential, and to rule out sharing of non psychoactive medicines. All interviews were carried out using a topic guide. Questions explored issues around current prescription drugs of abuse, drug seeking behaviour, the role of diverted pharmaceuticals, impact of prescription drug misuse and PHCPs' response to the behaviour within the primary care setting. Challenges faced by PHCPs are described elsewhere [11].

Note that the terms 'prescription drug misuser' and 'drug seeker' are used interchangeably throughout this paper to denote a patient involved in misusing psychoactive prescription medicines. During interviews, the researcher adopted the terminology utilised by the interviewee.

The research received ethical approval from the University of Auckland Human Participants Ethics Committee.

## Results

Fifty one semi-structured interviews were undertaken with GPs (n = 17), CPs (n = 16) and KEs (n = 18). Interviews last between 25 and 75 minutes. The sample included six female GPs and 10 female CPs. Nine of the GPs interviewed were authorised to prescribe methadone, and 13 CPs were involved in dispensing the drug as part of methadone maintenance treatment. Seven GPs had been practicing for more than 20 years, with three having been employed as a GP for between five and nine years. Three of the CPs had been practicing for less than five years, and ten had been doing so for more than 20 years. In addition, four CPs and five GPs were based in rural locations. KEs from areas including drug treatment, health or drug policy, law enforcement, and PHCP representative organisations took part. Four also worked as GPs, thus enabling them to comment on PDM from both perspectives.

The main types of prescription medicines identified in interviews as being misused were opioids (morphine, dihydrocodeine, codeine and pethidine), benzodiazepines (e.g. diazepam, clonazepam, temazpam, and triazolam), stimulants (e.g. Ritalin™) and other medicines such as zopiclone.

Part of the research explored interviewee perceptions regarding the type of people involved in misusing prescription medicines. During the initial stages of data collection, this was elicited via an open-ended question: *who are the main people involved in the misuse of prescription medicines? *Where necessary, further probing was undertaken in specific areas, including such patients' age, gender, ethnicity and socio-economic status. It was not intended to obtain a quantitative demographic profile of prescription drug misusing patients; rather, we were interested how PHCPs defined the characteristics of this patient group, and the qualities they were ascribed.

Findings revealed that, qualitatively, there was no unified picture of the 'typical' drug seeker in terms of their demographic profile. Indeed, interviewees were often quick to point out that it was difficult to generalise about patients who misuse prescription drugs as they came from *"all walks of life"*. PHCPs were, however, categorising drug seekers in other ways. This was primarily based on their views of patients' reasons for seeking illicit supplies of prescription drugs, how they came to start using such substances, and their relationship with prescription medicines. This distinction was also made by some 'key experts' who took part in the research. These interviewees tended to either be also working as a PHCP or were employed within the drug treatment sector.

Two key 'typologies' emerged from analysis of the data. For the purpose of this paper, they have been given the titles of 'abusers' and 'over-users', and a description of each is provided below.

### 'Abusers'

This first group of patients, whom we have called 'abusers', were the most strongly linked with prescription drug misuse and most interviewees, when considering the type of patients involved in this behaviour, initially attributed them with the following characteristics. 'Abusers' were believed to acquire prescription medicines for their own use or for selling on to the illicit market. They were either viewed as 'recreational' drug users who sought prescription drugs for the 'high' that they provided, or as 'addicts' who used them to knowingly feed an addiction. It was generally believed, therefore, that the prescription medicines obtained by these individuals were never used for their 'medically' recognised function, and that obtaining them from primary care was a deliberate act of deception.

'Abusers' were perceived as having a history of drug misuse, considered likely to be polydrug users, and with co-existing mental health issues. This included methadone patients or individuals known to be receiving treatment from specialist alcohol and other drug services. They were also typically believed to be younger patients, and more closely aligned, although not exclusively, with seeking pain-relieving drugs or stimulants (e.g. methylphenidate) rather than benzodiazepines.

It was believed that some patients in this category misused pharmaceuticals in a recreational fashion, and to derive some form of pleasure. Interviewees spoke about them using the substances for the '*high'*, the '*buzz' *and to have some kind of '*trip'*. In line with this was the implication that 'abusers' have some kind of control over their drug use, and that it is a conscious decision on their part to become intoxicated:

*You've got the other group *['abusers'] *who are addicts, who are coming off say 'p' *[street name for methamphetamine] *or are, you know, methadone clients. They use it for a buzz, they get a bit of a buzz off it. They have a little party and they drop it at the same time... yeah yeah, they'll save them up and then they'll drop them all on a Friday, and then over the weekend if they've got diazepam or something*. [KE]

It was evident that when discussing 'abusers' GPs and CPs sometimes merged their views of these patients with more general opinions of illicit drug-using patients. Indeed, GPs and CPs often subscribed to the unsympathetic depiction of drug users (and by association, 'abusers') as less than desirable members of society. Comments about the way in which 'abusers' looked (usually described as 'scruffy' or 'dishevelled'), their work situation (generally unemployed) and their lifestyle ('transient' or 'with no fixed abode') all served to reinforce the 'junkie' stereotype [11]. This was evident in an interview with a community pharmacist who spoke about how she identified individuals who were misusing prescription drugs. For this practitioner, 'abusers' are positioned as the 'bad guys' who have little to offer society and are seen as being 'abnormal' in some way.

*The down and outers and the pathetic stories and now they are pretty clever with being sort of looking normal and telling better stories I guess... Usually it's the ones that, what would you say, the real down and outers. You know, they haven't got hope, they'll be shoplifting as well and, you know, they're probably in and out of jail*. [CP8]

The stigmatisation of drug users by PHCPs was also raised as an issue during interviews with 'key experts', particularly in relation to the potential for this to impact on how prescription drug misusing patients were viewed:

*You know, the way that drug users are portrayed in the media and some of the comments you actually even get from within the alcohol and drug sector about drug users, you know, you kind of get this whole sense that it's kind of their own fault, that they're dirty people. I still think that's an understanding out in the community and I don't think doctors are immune to that stereotype*. [KE6]

It is important to note that not all interviewees expressed unsympathetic portrayals of illicit or prescription drug misusers. Moreover, there was evidence that some were aware of their 'biases' as evident in the following interview extract:

*It's like, I don't know, this sounds real mean, drug users like they're really skinny and really pale and got like tattoos. That's really bad, but they've got tattoos. There's just something that you just can pick them. Don't ask me why, like you just know after a while*. [CP9]

### 'Over-users'

This second group of drug seekers were not normally discussed straight away. Indeed, it was often only later in an interview that PHCPs remarked on a different category of patients involved in drug seeking behaviour. Some were even unsure as to whether or not 'over-users' should be classified as prescription drug misusers despite meeting the defined criteria for the behaviour.

Patients categorised as 'over-users' were believed to have begun using prescription medicines in a legitimate fashion. Interviewees spoke about these patients having an initial health issue, whereby they had been prescribed medication (e.g. pain relief) to manage the problem. The misuse behaviour had, therefore, only come later, and there was the implication that it would never had occurred in the first place if the medical condition had not been present. In line with this, it was believed that 'over users' sought prescription medicines for their own use only, and were not involved in selling their supplies on the illicit drug market, or to other drug users. It was also assumed that these patients were non-users of any illicit drugs:

*Yes, they *['abusers'] *have started and developed, particularly their opiate habit, through using drugs recreationally. Whereas the prescription patients *['over-users'] *usually would have had something like, particularly the younger ones, a road traffic accident or an injury at work, which has caused them to be put on to an opiate initially. So, they may well not have been a drug user at all*. [KE14]

It was commonly believed that the misuse was, in part, the fault of errant GPswho prescribed potential drugs of abuse over long periods of time, without appropriate checks in place. Thus, some interviewees felt that the medical profession needed to take some responsibility for the development of the misuse behaviour. Inherent in all of this was a sense that these patients were somehow transformed into prescription drug misusers through no fault of their own and, in some instances, without any self awareness that this was occurring. This medical basis for their addiction was somehow more acceptable and garnered greater empathy than that of the 'abusers':

*I mean I have a patient myself who's on morphine that started in the hospital and now, you know four or five years later she's still taking them and there's no way she's ever going to get off it. You know, we've tried, she's been under the pain clinic and, you know she's basically a drug addict at the hands of the medical profession. And you know, we have to take some, sometimes we have to take some blame for these things starting*. [Interview GP1]

There was evidence of some judgement being made with regard to the type of effect 'overusers' sought from their prescribed medication. Interviewees spoke about the substances being used by 'overusers' to *'feel normal' *or *'at peace'*, or in a functional way to stave off the effects of withdrawal. Compared with 'absusers', there was no association with the drugs being consumed recreationally or 'for fun'. In the following excerpt one GP is responding to a question about whether a particular patient (who she viewed as an 'overuser') would be using the prescribed drugs differently to patients who she classified as 'abusers', and she highlights what she sees as the differences between the two 'types' of patients.

*The drugs she's *[an 'overuser'] *using are not necessarily quite as, they don't give the same, they're psychoactive but they're not psychoactive in a way that you and I would want to trip or think that they want to trip or something. Whereas the other people *['abusers'] *come and they want their benzos, they want their morphine, we know that they're after a trip on it. Whereas the other people are desperate to maintain some sort of I don't know, whether they're turning their heads to some sort of peace, I don't know*. [GP6]

Long-term users of benzodiazepines, patients 'inherited' from another prescriber, and older patients were often categorised as 'overusers'. Indeed, it would appear that demographic characteristics sometimes played a role with regard to how drug-seeking patients were perceived. In the following account, a GP is debating whether a patient could be considered to be a drug seeker, despite exhibiting classic signs of 'doctor shopping' activity, whereby more than one doctor is visited in order to secure supplies of a potential drug of abuse. Whilst recognising that this was going on, the age of the patient - in their late seventies - clearly makes the interviewee question whether or not they could be seen as a prescription drug misuser. This would suggest that such behaviour is still considered to be the domain of young people and may mean that older patients are overlooked as potential drug seekers:

*Well, I mean I would generally say I think it's younger people and I think it's probably all ethnicities and both genders *[who misuse prescription drugs]. *I mean I suppose there are elderly people that drug seek. Well, it's drug seeking in a different way. Like I had a patient a few years ago, she was 79 or something. I gave her Gees linctus, which has a sort of opiate base...anyway, she'd never had it before and she came back three weeks later and said she still had a bit of a cough and so I gave her some more. And then she came back - oh she saw a colleague of mine a few weeks later and got some more, and then she came back to me and asked for more and I realised. And she'd become addicted to it - so I don't know if you call that drug seeking? *[GP3]

### How do these constructed identities impact on the way in which PHCPs respond to prescription drug misusing patients?

The first part of this paper has described two identities ascribed to prescription drug misusing patients. This section will explore how these constructed identities or typologies were reflected in the way primary healthcare practitioners responded to drug seeking behaviour either within their practice (in the case of GPs) or in the community pharmacy setting (for CPs). Specifically we focus on whether or not GPs and CPs offered some form of harm reduction intervention to such patients, and if so, how this was shaped by the way in which the patient was categorised. Interventions within this setting could include providing information to patients on the health effects of misusing potential drugs of abuse, offering general help or assistance (e.g. trialling 'drug-free' days) or referral to a specialist service (e.g. drug treatment or a pain clinic).

#### Tom and Jerry

It is worth noting in the first instance that under their respective professional codes of conduct, GPs and CPs have professional and ethical obligations to prevent the misuse of medicines. In line with these responsibilities, the predominant response (by both CPs and GPs) when faced with an incident of PDM involved attempts to control the supply of medicines. In general, this involved ensuring these medicines were not made available, by refusing to prescribe them (GPs) or dispense them (CPs) or limiting the amount provided. Other strategies included banning the patient from the practice or pharmacy, and either contacting Medicines Control staff or Police. PHCPs sometimes varied their response depending on the circumstances (e.g. if they felt threatened) or the nature of the therapeutic relationship (e.g. if it was a patient they had been engaged with over a long period).

Nonetheless, descriptions of the way in which health professionals engaged in this policing of the system provides evidence of the typologies they attributed to the prescription drug misuser. The positioning of the 'abuser', for example, as someone trying to swindle the health system and whose access to prescription medications needs to be prevented, conjures up something of a 'cat and mouse' scenario, with the GP or CP attempting to stay 'one step ahead' in order to 'catch them out'. One GP acknowledged that *"it's sort of like a competition amongst some of the more senior docs to know whether they've been done or not"*. Findings from the research suggest that this dynamic has the potential to overshadow the fundamental healthcare role of PHCPs, with the focus on not being 'caught out' or 'duped', rather than the management of potential health and other risks to the patient involved in the activity.

In contrast, the refusal to supply prescription medicines to the 'abuser' and the emphasis on not being deceived by them, was not necessarily seen as an appropriate response to the 'overuser'. Indeed, health professionals might even actively avoid any legal or regulatory sanction when dealing with the 'overuser'. One GP, for example, recalled her concerns around a patient for whom she was prescribing high levels of Halcion™ (triazolam) and Imovane™ (zopiclone). She described him as being *"chronically addicted to benzos"*, was uncomfortable continuing to prescribe to him at the same level, and thus considered contacting a regulatory body to seek assistance in monitoring his use. In the end, however, she decided against this course of action due to the individual being (what she considered) an 'over user' rather than a typical drug seeker (i.e. an 'abuser'):

*But I didn't do it *[contact Medicines Control] *for that guy in the end because I think that he's actually not a drug seeker. Well he's a drug seeker in that he's totally addicted to these things but he's not, I don't believe he's passing them on or using them for any purpose other than to manage his day-to-day back pain*. [GP3]

#### Different strokes for different folks

Less commonly reported were attempts to support patients, to make referrals to treatment services and to instigate harm reduction interventions. Moreover, where interventions were undertaken, the nature of these was clearly shaped by the way in which patients were perceived.

The stigmatised identity of the 'drug addict' patient (i.e. those classified as 'abusers') became an issue for some PHCPs in situations where there was the potential to offer some kind of intervention. In keeping with the belief that 'abusers' were somehow to blame for their own demise and undertook their drug seeking in a more calculated fashion, some interviewees expressed less empathy for this group of patients, which carried over into their therapeutic responses. The following excerpt is one GP's response to being asked about how he would manage a patient who he considered to be an 'overuser':

*Yeah, in a supportive way, absolutely*. [GP11]

This is contrasted with his response to patients he considered to be 'abusers':

*Well the drug seekers *['abusers'] *are taking advantage of you, they're liars and manipulators. And whereas, you may have a relationship with a patient *['overuser'] *who you might inherit a patient from someone, or a new patient who comes with the warmest recommendation of their previous GP and an admission that, you know, they do have this problem as a result of an accident years ago and yes they are on ... oxycodone say. I've got a patient that's on oxycodone that uses a lot of it for a terrible bowel problem and he sees a top surgeon in town regularly. He's addicted to the stuff but, you know, so what? You know, I do everything I can to help him... once you feel that this person is genuine, not manipulative, not using you for advantage, then of course, you know, the doctor in you comes out and you help them as much as you can*. [GP 11].

A community pharmacist described how their response to prescription drug misuse would vary, depending on how they viewed the patient's behaviour. In the case of 'abusers', they reported that they would involve the police, whereas they had previously undertaken some kind of harm reduction intervention with patients in the 'over users' group:

*I think probably it's how do we determine it's abuse and not overuse and probably I tend to help the overuse - if I think it's overuse - as opposed to the abuse, which I will ring the cops or if it's a forgery. Yeah because I have, I have a patient now who is on weekly dispensing who we got involved with TRANX [*a drug treatment service that specialises primarily in benzodiazepines addiction/dependence] *because she was overusing so it is abuse but I don't think she's abusing it for the psychoactive effects. I think she was just overusing it for her own, just trying to cope*. [CP2]

The way in which drug addiction itself is positioned as something shameful and either hidden or unknown is also evident in one community pharmacist's discussion as to why they had rarely undertaken any harm reduction interventions with patients who they believed to be misusing prescription drugs. Of particular note is the way in which she described how such a patient might be approached - i.e. that they would be *accused *of having a drug problem. It is interesting to contrast this with other health issues, such as diabetes or angina, where it is difficult to imagine that health care professionals would consider these conditions in the same way, and be anxious about 'accusing' a patient of having such a health issue:

*I don't think it *[harm reduction interventions] *really happens here but I certainly think there is the potential for us to play a part in that. I'm just not really sure how you would go about doing it or how it would happen really. It is a bit of a hard one, I think we do in a way have a responsibility for that, or to assist in that but it's how to get it received without causing a problem, without them ever turning very aggressive on you. It's - I don't know that's quite as easy as doing that. If someone asks, you know, if your customer asks for information or instigates it, then it's very easy to give information across. But for you to, you know, basically accuse them of them having a drug problem, it can be quite hard to instigate*. [CP16]

Also evident from this account, in which the pharmacist acknowledges that there is a duty of care to offer harm reduction interventions, are a number of reasons for this not occurring, including a lack of knowledge as to how to go about it, and a fear of *"them" *becoming aggressive.

Concerns over the way in which patients might react were not restricted to 'abusers'. Some interviewees indicated that they expected 'over users' may also respond negatively to a GP or CP-initiated intervention. The reasons for this, however, were somewhat different. One GP highlighted that patients in the 'over-user' category may not consider themselves to have a drug-related problem and in the following extract describes how this can make things difficult for the healthcare professional who is attempting to intervene and instigate some form of behaviour change:

*Others, like that little old lady, for some reason you get them going on them *[benzodiazepines] *because it's not as if you can never prescribe them because they're quite good drugs. And then they find them helpful and then it's quite hard to talk them out of it because there's lots of people that actually don't see that argument long term you'll become addicted and need something every night and the side effects that, you know, you're less well, you're less crisp, your concentration is poor, you can fall more. But you get the counter argument, but doctor I can't sleep and if I can't sleep I fall more and I've got poor concentration and you know, you sit there and it's quite hard to actually justify not giving them something that does sound really helpful. You know, so it is a very difficult area, very difficult. [GP3]*

Another GP questioned whether intervening was necessary, or even appropriate for these patients:

*I mean some years ago there was a preponderance of middle aged and older people being on them *[benzodiazepines]. *And so that's probably a bit less common now but with some of the older ones who have been on them a while who are resistant to coming off you might think, well, you know, if they've been on them this long and they're going to die in a few years, why bother getting them off? *[Interview GP9]

#### Getting to know you

For the most part, GPs and CPs spoke about having longer term relationships with 'over users', given that they were often elderly individuals who had been linked with the practice or pharmacy over a period of years. In contrast, 'abusers' were frequently 'one off' patients who attempted to secure illicit supplies of prescription drugs and, when unsuccessful, were likely to leave the premises quickly, rarely to be seen again (although there were some exceptions).

The practicalities of undertaking some kind of harm reduction intervention with a drug seeker unknown to the practice or pharmacy, were highlighted as potential barriers by some interviewees. As evident in the following interview extract, it was not seen to be feasible where patients were keen to spend limited time in the consultation and it was expected that they would not be interested in accessing any help:

 I: So do you think there are any opportunities for GPs to get involved in harm reduction in this way?

R: ... *Not for the one off drug seeker that comes into your office, you know, they're not going to, the reality is that they're not going to break down and say, 'oh yes doc, you're dead right and I'm hopeless and give me help'. You know, they're there with an agenda and they're moving on and they'll be new to the area. If they're not going to get the goods they're out of there*. [GP11]

Alongside the obvious practical difficulties of instigating a brief intervention within a single encounter with a new drug seeking patient, the lack of a relationship with a patient had other implications. One GP, for example, felt less inclined to help casual patients:

*I guess if they're a casual patient and coming in seeking some obvious substance, then you know, you're quite blunt with them and send them on their way. But if it's a long term patient who you've developed a relationship with you try to sort them out better. And try and yeah I guess I manage things a bit differently, rather than just send them on their way*. [GP15]

#### 'Over users' falling through the gaps

In general, drug misuse is a covert activity, possibly only being revealed within one's social networks. It was acknowledged that 'over users', however, may be less likely to share their substance use problem. One key expert highlighted that, for the 'overuser,' this could mean they had no access to a support network and subsequently less help in relation to their prescription drug use:

*Often with the illicit drug use *['abusers'] *it's the whole culture and group of people using illicitly together, so there's discussion about the use within that group, peer group. And there's support within that, and there's sort of 'you're getting in trouble here', or 'go there, you'll get something there'. If it's people who have come through the other doorway and have built up a dependence from getting drugs prescribed by their GP *['over users'] *they're usually quite isolated. They're not going to come to talk to their families about 'I needed three more sleeping tablets last night'. I think that would be unlikely so I think that group are alone a bit more. And probably less likely to know where to go for help... they're a naive user really*. [KE10]

In line with this, it was considered that over-users would not see themselves as 'addicts' or as a part of the drug-taking cultural milieu. Thus, even where practitioners were willing to engage in harm reduction interventions with 'overusers' (e.g. referral to a treatment agency), findings from the research indicate that practitioners believed that this group of patients may not view traditional treatment options available as relevant or appropriate:

*I think for some, you know those two groups again, depending on if someone's using other substances and seeking the drugs to support the other drug use *['abusers'], *they belong with NA *[narcotics anonymous], *but the group who may have unintentionally ended up with a dependency *['over users'], *may not see that they fit with that illicit culture*. [KE10]

#### The value of experience

Despite much of the data pointing to a potential lack of engagement in harm reduction interventions with 'abusers', there was evidence that practitioners with a different mindset, with prior training and experience, and working in situations where no other treatment was available, might be willing to tackle the issue. One experienced GP with extensive exposure to drug-using patients and training in the area of drug misuse (he was also authorised to prescribe methadone and had a large methadone patient base) describes below his approach to managing his prescription drug misusing patients:

*....Because you confront the addiction factor of it and start to say, 'well look okay this is realistic' and 'what are we going to do about your addiction' and not 'what are we going to do about you not having this prescription?'. So you see it as a problem and a health related problem and you start to become more realistic around genuine interventions*. [KE5 and practicing GP]

This same GP went on to highlight the nature of his therapeutic approach:

*When you're a rural practitioner you're a monopoly provider and there is a, if you like, an ethical obligation to be therapeutic for everybody - they don't have another option. You can't just say "piss off noddy because you're annoying me" because that person still has health needs and will still need to access my service on an ongoing basis and for other reasons. So you tend to try and take a therapeutic approach in the first instance and say,'look I think there's an issue here - you're either addicted to these drugs or you're abusing them, one or the other'. So I confront the patient with the issue, 'what are we going to do about that?' And I put the onus back on them and some people will respond to that and others won't, and others will walk or storm out and abuse the receptionist on the way past, whatever they choose to do*. [KE5 and practicing GP]

## Discussion

This paper has drawn on the findings from a research study which explored the issue of prescription drug misuse within primary care. It did not set out to specifically investigate whether or not PHCPs viewed drug seekers as a homogenous group - this was something that was identified during data collection, and explored further as part of the analysis process. The findings reveal that perceptions were of two distinct groups of drug seekers who were viewed quite differently and often elicited distinct (and often opposing) responses from PHCPs. Whilst much has been written about practitioners' attitudes towards drug misusers per se, and their treatment within primary care settings [12-15], we have found little which has examined this issue within the context of patients involved in misuse of prescription medicines, specifically. A small-scale study undertaken in the UK which explored views of high-dose benzodiazepine-dependent patients also identified that these individuals were not considered a uniform group, with distinctions made between housewives "with anxiety problems" and polydrug users [16].

When discussing drug seekers, most of the dialogue centred on the group perceived to be 'abusers', and there were clear indications of greater levels of empathy with 'over-users'. Indeed, the research has provided further evidence of the way in which drug addiction is highly moralised, and has shown that primary healthcare practitioners are not exempt from this. This is perhaps not surprising given the widespread stigma and marginalisation experienced by drug using members of society [17,18]. In our study, there was much evidence of stereotypical views held of prescription drug misusers as 'addicts', with associated, often negatively portrayed, lifestyles and appearance. The stigmatisation of drug users within primary care settings has been widely discussed in the literature [19-21]. A study which investigated the reasons why some community pharmacists were reluctant to provide services to drug users revealed a lack of approval by staff or customers, a potential increased level of shoplifting by patients accessing the service, and business reasons as being the basis for this [13]. Research undertaken with GPs identified that the majority of GPs interviewed held at least some negative views towards drug users. This generally related to patient behaviour (e.g. missing appointments) or due to threats to safety. However, whilst the authors note that 'difficult' and 'manipulative' were commonly used terms with regard to these patients, there was also evidence of more positive and accepting attitudes amongst some GPs [22]. In addition, the way in which GPs and CPs respond to the issue, may, in part, be influenced by the degree to which they believe they have contributed to the behaviour by historically having facilitated or enabled acquisition of prescription medicines.

A lack of training in the area of addiction and/or substance abuse has been identified as contributing to stigmatised views amongst health professionals of drug using patients, or an unwillingness to undertake harm reduction interventions such as counselling [19,23,24]. In overcoming some of the stigmatised views held by PHCPs, we would assert that training and education need not be complex nor resource-intensive. Fairly simple activities such as undergraduate medical and pharmacy students receiving talks from ex-prescription drug misusers may serve to de-mystify substance use and challenge some of the pigeonholing and negative labelling that occurs. It is, however, also worth considering that education and training on its own, is not likely to be enough to shift negative attitudes towards drug users/prescription drug misusers. In their review of research on the attitudes of health professionals towards alcohol and other drug (AOD) work, Skinner and colleagues highlight that organisational culture plays a role in this issue, alongside a health professional's personal standpoint on drug use and matters of social justice [15].

Within the context of prescription drug misuse specifically, the findings from this study have some important implications. Firstly, patients who misuse prescription medicines (particularly those deemed to be 'abusers' by their GPs and CPs) may be stigmatised in the same way as illicit drug users in general. There was evidence of a lack of empathy in relation to the personal circumstances of 'abusers', with their addiction seen as being their own fault, able to be controlled, and something that they chose to do. It is possible that this may also impact on a patient's care in relation to other areas of their health. Baldacchino and colleagues, in a study of chronic non-cancer pain management of patients with a substance misuse history, also noted that physicians indicated that their judgment of a patient with a substance misuse diagnosis might adversely impact on the patient's pain care [25].

In many cases, PDM was viewed as a legal matter rather than a health issue. This is in line with previous research from the US which explored the knowledge and attitudes of pharmacists towards prescription drug abuse. Half the sample saw their position as incorporating both a policing role as well as a healthcare professional, and when they were asked how prescription drug abusers should be treated - as patients with brain disorders, as people with illegal behaviours, or as both - nearly three quarters indicated 'both' [24]. There is clearly a tension between a health professional's need to work within their scope of practice and adhere to the codes of ethics and guidance provided by their regulatory bodies, and a desire to provide help and treatment for those with problematic substance use. It may be that in classifying those who misuse PDMs in the way our respondents have, they are seeking to legitimise or justify their responses to the issue.

Secondly, given the dominance of the 'abuser' typology, it is probable that primary healthcare professionals may overlook some drug seeking individuals who fall outside of this image. Thus, those patients who are well-presented and articulate may not be considered potential misusers despite exhibiting suspicious behaviour (e.g. specific requests for a potential medicine of abuse). At the same time, 'scruffy', tattooed individuals may be unfairly suspected of misuse behaviour, and possibly denied legitimate treatment. Clearly, PHCPs need to be aware of their own internal judgments and preconceived ideas of drug misuse and prescription drug misusers.

Similarly, how patients were categorised clearly influenced the way in which some PHCPs responded to incidents of PDM. Interestingly, there was evidence that the responses to 'overusers' (particularly long-term users of medicines such as benzodiazepines) could be inconsistent. On one hand, practitioners indicated that 'over-users'' misuse problem may not be addressed due to the perceived lower level of harm (to self and society) associated with this type of PDM. Conversely, it is possible that these patients may receive a greater level of care, given the sense of compassion that was expressed towards their problem - particularly in cases where their dependence/addiction was considered iatrogenic.

It notable that it is not only health professionals who may stigmatise drug users, but also drug-taking individuals themselves [11,26]. Research with problematic drug users in the UK found that some users rejected the "junkie" identity commonly associated with criminality and un-controlled heroin use, and were careful to distinguish themselves from this stigmatised identity and other drug users who they categorised in this way [11]. This is in keeping with the view of some healthcare practitioners in our study that referral to a traditional drug treatment centre may not always be appropriate for patients who misuse prescription medicines. It would also be interesting to conduct further research with prescription drug misusers themselves and explore whether such typologies do indeed exist - and whether or not the two types of patients described in this paper express similar views to those of primary healthcare practitioners.

Finally, as with all research, our study is not without its limitations. The research was conducted in New Zealand, which has a particular illicit drugs market and high reliance on diverted pharmaceuticals. The view and practices of PHCPs who might have been involved in over-prescribing or inappropriate supply of prescription medicines may also not be represented here.

## Conclusions

This study has uncovered two typologies of prescription drug misusers, as described by PHCPs, and has explored the potential associations between these typologies and health practitioners' engagement in harm reduction and treatment interventions. Results from the study indicate a need for further exploration of these issues, in particular 'over users' whose needs may not be being met by mainstream drug services, and issues of stigma in relation to 'abusers'.

## Competing interests

The authors declare that they have no competing interests.

## Authors' contributions

JS conceived of, and designed the study, was involved in the analysis and writing of the paper. RB carried out the data collection, undertook the analysis and drafted the manuscript. All authors read and approved the final manuscript.
